# The influence of parents gender attitudes on students career choices: a sociocultural analysis

**DOI:** 10.3389/fsoc.2026.1845411

**Published:** 2026-06-09

**Authors:** Abikenov Zharkynbek, Zhuzeyev Serikkhan, Syzdykova Makhpal, Abdiramanova Aigul, Kudaibergenov Sergazy

**Affiliations:** 1 Korkyt Ata Kyzylorda University, Kyzylorda, Kazakhstan; 2 Al-Farabi Kazakh National University, Almaty, Kazakhstan

**Keywords:** career choice, cultural transformation, gender, sociocultural analysis, survey research, theoretical analysis

## Abstract

**Introduction:**

In today's world of globalization and changing labor markets, students' career choices are directly influenced not only by their individual abilities, but also by the socio-cultural environment of their families. The main goal of this study is to investigate the mechanisms through which parents' traditional gender beliefs influence students' career decisions in Kazakhstani society from a sociocultural perspective.

**Methods:**

The study used a cross-sectional quantitative design and involved 701 university students. The research hypotheses, which state that parents' traditional gender attitudes are positively associated with the level of interference in student choice, were tested using Pearson's correlation, analysis of variance (ANOVA), and linear regression methods.

**Results:**

Empirical analysis confirmed a significant positive relationship between traditional parental attitudes and their directive intervention (*r* = 0.427, *p* < 0.001). The regression model explained 18.2% of the variance in parental influence (β = 0.427). The results showed that gender segregation in career orientation is shaped by economic pragmatism, cultural dogmas, and implicit pressure from parents. Students' choice of professions that “do not match” their gender (e.g., STEM for girls) leads to less psychological support from their families.

**Discussion:**

These findings suggest that young people's career decisions are more influenced by “social approval” than by individual abilities. Career choice is a complex socio-cultural process influenced by family. To overcome gender inequality in the workforce and fully utilize human capital, it is not enough to focus only on students. We need to shift to a “family-oriented” approach to professional counseling. This approach aims to deconstruct gender stereotypes in parents' minds.

## 1 Introduction

In the context of contemporary social sciences, the impact of parental gender attitudes on students' career choices has been identified as a significant area of research. With the rise of globalization, the transformation of education systems, and changes in the job market, professional choices are influenced not only by individual abilities and interests but also by socio-cultural factors.

Among these factors, parental influence is particularly significant, as the family serves as the primary socialization environment for individuals and plays a crucial role in shaping their values, worldviews, and professional orientations.

Gender stereotypes are passed down through parents' expectations and attitudes toward their children, and can directly or indirectly influence students' career choices. In particular, the traditional division of professions into those considered more profitable for “men” (engineering, IT, management, industry) and those considered more family-friendly and stable for “women” (pedagogy, healthcare, service) persists. These stereotypes lead girls to focus on fields that are more compatible with family life and socially approved, rather than on technical (STEM) fields.

To form the conceptual basis of this study, we prioritized several fundamental theories that explain the phenomenon at the macro-social, family, and individual cognitive levels. First and foremost, the origins of parental attitudes toward career choices are linked to the transformation of macro-social values. To this end, Inglehart's (2005) theory of modernization and Schwartz's (1992) theory of human values are taken as a foundation. These macro-level changes, as cross-cultural research has shown, determine the specific cultural context of each region, including the nature and intensity of intergenerational career orientations.

Bourdieu's theory of social capital and habitus (1986) is used to explain how global and cultural values are transmitted through the institution of the family. In this context, career choice is not just a psychological preference. The cultural capital provided by the family, as well as the established traditional gendered habitus, unconsciously determines the precise boundaries of a student's professional opportunities (Tandrayen-Ragoobur and Gokulsing, 2021).

Cultural capital can be divided into two main types: embodied and objective. Embodied cultural capital refers to the skills, knowledge, and abilities that individuals possess, while objective cultural capital refers to material goods such as books, art, music, and other cultural artifacts. Additionally, there is institutionalized cultural capital, which refers to the possession of higher education degrees or diplomas (Bourdieu, 1984).

At the micro-level, that is, in the process of personal decision-making, gender and cognitive factors play a significant role. According to Bem's (1981) gender schema theory, the ability to categorize information into male and female categories, acquired at an early age, indirectly influences young people's choices. The social cognitive career theory, based on Bandura's (1986, 1997) social cognitive theory and Roe's (2008) theory of career choice, explains how family pressures and norms shape a student's personal preferences. Parental parenting styles directly influence career choices, shaping a child's sense of self-efficacy, professional motivation, and ultimate goals (Lv et al., 2022).

However, most research on this topic has been conducted in Western societies. In Kazakhstan, the influence of parental gender attitudes on student career choices has not been thoroughly investigated. Given the region's sociocultural characteristics and the preservation of traditional values, it is necessary to conduct a more in-depth analysis of the mechanisms underlying parental influence (Brussino and McBrien, 2022; Morrow, 2018) Additionally, the relationship between the transmission of gender stereotypes through parental influence and students' autonomy has not been comprehensively explored (Fadlelmula et al., 2022; Haunberger and Hadjar, 2020).

In a society that places great value on parental authority, the concept of “autonomy” rarely translates into legal freedom to choose a career. Rather, it is more about the psychological feeling of independence, and whether students feel genuinely free to follow their own interests or subconsciously align their choices with internalized family expectations in order to maintain harmony.

In this study, we aim to identify the mechanisms through which parental attitudes toward gender influence students' career choices. We will examine the specific ways in which gender stereotypes are transmitted through parental influence and the role of individual autonomy in students' decision-making processes. This research is of significant pedagogical importance, as it can contribute to a better understanding of the complex relationship between family, education, and career guidance.

To achieve the main goals of the study, we aim to answer the following research questions:

1. How do traditional gender roles and attitudes of parents influence the level of parental involvement in a student's choice of profession?
2. To what extent do gender stereotypes within the family limit students' (especially female students') autonomy in choosing STEM (science, technology, engineering, and mathematics) and humanitarian fields?

Based on the theoretical concepts presented in the study and the results of cross-cultural analysis, we propose the following research hypotheses in the socio-cultural context of the southern regions of Kazakhstan:

1. As parents' traditional gender views become stronger, the level of their involvement in their children's career choices increases.
2. Parents, despite the demands of the modern labor market, tend to direct their children toward careers that are safe and consistent with traditional gender roles.
3. Traditional gender positions in the family more severely limit the autonomy of girls in choosing a career (especially in technical/STEM areas) compared to boys.

## 2 Literature review

To establish the conceptual basis of this study, we prioritized a system of fundamental theories that comprehensively explain the process of career choice, from the transformation of macro-social values to individual cognitive decision-making. This review analyses the interactions among parental influence, gender stereotypes, cultural norms, family social capital, and personal psychological factors.

Parental attitudes are influenced by the system of personal and cultural values. In this regard, Schwartz's theory of human values plays an important role in understanding career choices and family influences (Schwartz, 1992).

In Schwartz's (2012) model, parents who prioritize “preservation” values see their children's future through the lens of safety and conformity to societal norms. These value orientations restrict family members' risk-taking and often lead children to pursue traditional, socially acceptable gender roles.

This micro-level phenomenon aligns with Inglehart and Welzel's (2005) theory of modernization at the macro-social level. Their research suggests that societies shift from “survival” values to “self-expression” values, and from traditional to secular-rational values, as they become more economically and socially developed.

In transitional societies like Central Asia, modern educational trends coexist with traditional patriarchal values (Inglehart and Baker, 2000; Inglehart and Norris, 2003). This duality of values is evident in the fact that while parents support higher education for their daughters, they limit their daughters' future career options to social or humanitarian fields such as pedagogy and medicine, and encourage their sons to pursue pragmatic, financially rewarding technical paths (Charles and Bradley, 2009).

This intergenerational transfer of values significantly restricts students' autonomy in choosing their career paths. Such limitations are not unique to this region; empirical evidence from other developing and traditional contexts demonstrates that deeply ingrained cultural norms act as significant barriers to women's occupational choices and distinctly shape their achievement motivation (Ghosh, 2021; Osiruemu, 2007).

Cross-cultural research has shown that the extent and nature of parental influence can vary significantly across cultural contexts. In Western countries, such as the Scandinavian nations, studies have found that overt gender stereotypes have decreased as a result of long-term government efforts to promote gender equality. However, research has also found that implicit biases persist in these societies, especially in STEM fields, as demonstrated by Charles and Bradley (2009).

An important direction in modern gender theories is the intersectional approach. This concept, introduced by Crenshaw (1991), underscores the need to consider gender inequality alongside other social categories (race, class, and ethnicity). This approach demonstrates that gender experience is not uniform and manifests differently across social groups.

The intersectional approach is particularly important in cross-cultural studies because gender norms in each society are shaped by the cultural context. This allows us to understand the complex nature of gender roles, especially in post-Soviet and traditional societies. Feminist economic theory is crucial in analyzing gender inequality from an economic standpoint.

Seguino (2007) argues that gender inequality is an integral part of macroeconomic processes. He believes that the division of labor, income inequality, and social policies play a crucial role in shaping gender relations. This perspective allows us to understand gender not only through cultural factors but also through economic structures. Furthermore, empirical studies demonstrate dynamic changes in gender attitudes.

Dotti Sani and Quaranta (2017) found, in their research across European countries, a gradual shift toward more liberal attitudes toward gender roles.

However, these changes are not uniform across all social groups. They depend on educational level, age, and social status, as shown by Dotti Sani and Quaranta (2017). Charles and Bradley (2009) link gender segregation in education to cultural and institutional factors. They argue that even in countries with high levels of gender equality, gender differences in career choices persist, indicating the influence of cultural norms, as noted by Smyth (2007).

The process of choosing a career is not just a matter of personal psychological preferences or cognitive decisions. It can also be viewed as a complex social phenomenon closely related to Bourdieu's (1984, 1986) theory of social capital. According to this theory, the main agents of transferring cultural and social capital are institutions such as the family. These resources enable upper-class parents to provide their children with a high-quality education and create opportunities for them to achieve high social status in the future. Furthermore, the transmission of these critical values and skills frequently occurs through learning beyond formal education, heavily relying on community and familial structures (Nordvall, 2022).

Within this transmission process, parents' gender attitudes, as part of their internalization of societal norms and values, unconsciously influence the boundaries of their children's professional aspirations, determining what is considered possible and impossible in a given society.

Students who grow up with a traditional gender identity, even if they have high academic abilities, may be more likely to reject careers that are not considered “natural” for their gender. Their family background and the worldview they have formed determine how comfortable or uncomfortable they feel in a certain professional environment (Bourdieu and Passeron, 1990).

Parental influence is exerted not only through direct guidance but also through indirect social and cultural mechanisms. This process is closely linked to family resources, social and cultural capital, and the school environment (Tandrayen-Ragoobur and Gokulsing, 2021). The level of education, economic status, and professional experience of parents can influence a child's career choices.

This sociological perspective complements Gottfredson's (1981) theory of career choice in psychology, which suggests that children and adolescents evaluate career options and form a “zone of acceptable alternatives” during career orientation.

In this process, first of all, professions that are not “sex-typical”, that is, those that contradict gender roles, are excluded from the list, regardless of their prestige or level of interest. Later, when making a real choice, young people are forced to avoid options that are inaccessible or socially unacceptable, sacrificing their true interests to gender norms and parental expectations (Gottfredson, 2002). Thus, gender stereotypes, instilled through family habits, act as the first and most significant filter that pre-emptively limit a student's professional choices.

Williams (2024) argue that changes in gender norms are closely linked to family structures. Their study shows that although support for gender equality has increased in modern society, traditional attitudes have not completely vanished; rather, they have hybridized.

Gender inequality is also evident in the education system. Legewie and DiPrete (2021) analyze the impact of the school environment on the formation of gender differences and show that educational institutions are among the mechanisms that reproduce social inequality.

Bem's (1981) gender schema theory plays a fundamental role in explaining the gender asymmetry in career choices and the influence of parents on them. According to this theory, from an early age, children learn to process information about the world in terms of the categories “male” and “female” through socialization within the family. These cognitive schemas, which are transmitted by parents, directly influence students' determination of which careers they consider “suitable” (Bem, 1981).

Recent research has shown that the cognitive schemas that parents transmit in everyday life directly influence students' determination of which professions are “suitable” for them. These schemas are internalized by children, who incorporate their parents' opinions, approvals, and restrictions regarding professional fields into their gender schemas. This process shapes young people's professional self-esteem and limits or expands their career choices (Weisgram and Diekman, 2017).

The gendered parenting model proposed by Endendijk et al. (2016) suggests that parental expectations based on gender are important in shaping children's behavior and values. The use of different parenting strategies for boys and girls contributes to the reproduction of gender stereotypes, as suggested by Endendijk et al. (2016). However, the current research has not fully explored the relationship between the transmission of these gender stereotypes through parental influence and students' autonomy. Some studies suggest that parental support can increase students' autonomy, while other studies suggest it can create dependence (Fadlelmula et al., 2022; Haunberger and Hadjar, 2020), suggesting that it depends on the nature and content of parental influence.

The impact of gender stereotypes on career choices is often explained through the lens of social cognitive theory. This theory suggests that environmental role norms and expectations directly influence an individual's career path by shaping their sense of self-efficacy and ability to perform in specific professions (Walby, 2005).

This is closely related to the career-choice theory proposed by Roe (2008), which emphasizes the role of the initial psychological environment and parenting style in shaping a person's career orientation. Parental support, expectations, and beliefs can directly influence a child's sense of self-efficacy, motivation, and interests, according to these theories (Lv et al., 2022).

Research has shown that parents' gender attitudes can have a different impact on children's decisions about their future careers, depending on their gender. For instance, traditional gender stereotypes may reduce girls' interest in technical fields such as engineering (Chan, 2022).

During adolescence, social expectations about gender roles can influence career choice. Parental support can act as a buffer, helping children build their self-esteem and set career goals (Kelly, 2016). This support helps children develop their interests and overcome challenges (Chan, 2022). Psychological factors such as self-confidence and interest also play a role in shaping a person's career path, especially when it comes to gender differences.

Similar studies have shown that parental support increases girls' interest in STEM fields, and in some cases also increases their likelihood of choosing humanities (Smith and Wood, 2020; Brussino and McBrien, 2022). This study demonstrates the context-dependent moderating effect of parental influence on career choices and suggests that a single explanation is insufficient to account for the process. The types of support provided by parents also play an important role in influencing a child's career decision-making. Emotional support, guidance, and resource support all have varying degrees of influence on the process of choosing a career. For example, studies show that parental support can increase the likelihood of girls entering STEM fields (Sundly and Galway, 2021). However, in some cases, parental gender bias can have the opposite effect and hinder the choice of a non-traditional major.

The study of cultural change in Kazakhstan, especially in its southern regions, reveals the complexity of the “local trajectory”. Domestic researchers have shown that the impact of globalization on traditional values is not a simple clash but a complex, hybrid transformation in public consciousness.

For example, Syzdykova and Abdiramanova's (2024) observed how traditional values are being reinterpreted in a new social context. This trend was further explored by Buribayev et al. (2025), who found that modern Kazakh youth are preserving their traditional values while also adapting to modern Western ideals such as personal achievement and self-actualization.

In turn, Kudaibergenov et al. (2025) demonstrate that these value shifts are not superficial but form a deep and structural basis for cultural change in modern society. These structural transformations directly affect individuals' daily experiences at the macro level. Abikenov et al. (2025) emphasize the significance of family discourse and reveal the role of cultural factors in shaping interpersonal relationships and public consciousness. Bolysbayeva et al. (2024), meanwhile, delve into the socio-psychological underpinnings of this process and explain how cultural and gender factors influence social behavior models in new contexts. They argue that adolescent professional choices often balance traditional parental expectations with emerging global labor market trends.

Despite the extensive theoretical background outlined above, there remain important gaps in the current scientific literature. This justifies the significance of this study:

1. Contextual gap: the vast majority of studies are based on empirical data from Western countries. In societies such as Kazakhstan and Central Asia, where traditional values and globalization coexist, the impact of parental gender attitudes on students' professional choices has not been sufficiently studied (Brussino and McBrien, 2022; Chen and Guo, 2024).
2. Mechanism of gender stereotypes: the mechanisms through which parental influence, such as direct pressure or indirect manipulation, transmits gender stereotypes and their long-term impact on student autonomy have not been fully understood (Fadlelmula et al., 2022).
3. Comparative analysis: the relative importance of parental influence in the choice between STEM (science, technology, engineering, and mathematics) and humanities/social sciences requires in-depth cross-disciplinary research (Takhar, 2016; Tandrayen-Ragoobur and Gokulsing, 2021).

Thus, the literature review suggests that career choice is more of a socio-cultural phenomenon at the intersection of family values and societal gender norms than an individual decision. This study aims to address this gap in the academic literature by systematically examining the causal relationship between parents' gender attitudes and their influence on their children's career choices.

## 3 Materials and methods

This study was a quantitative research project examining the influence of parental gender attitudes on students' career choices within a socio-cultural framework. The study employed a cross-sectional survey methodology, which allowed for a thorough analysis of participants' professional choices and family cultural orientations over a specific time period. Demographic and familial factors were considered cross-culturally to reveal the social and cultural aspects of the study. The study involved 701 students from higher education institutions in southern Kazakhstan.

Participants were selected through convenience sampling to ensure socio-cultural diversity typical of a transitional society. Respondents came from various educational backgrounds, representing the main spectrum of the modern labor market, including pedagogical (humanities-traditional), IT/STEM (technical-innovative), and social sciences. This selection allowed us to compare the influence of family background on career choices in different professional fields.

Data were collected using a specially designed and culturally adapted questionnaire. The study focused on two main variables (see Table 1).

**Table 1:** Research variables and their descriptions.

Variable	Identification	Description	Question number	Measuring scale
Parents' gender attitudes	PGA_mean	Describes parents' traditional or modern views on gender roles and professions	8 (PGA1–PGA8)	5-point Likert scale
Parental influence	PINF_mean	Describes the level of influence of parents on students' choice of profession	6 (PINF1–PINF6)	5-point Likert scale

The study considered two main variables: parental gender attitudes (PGA_mean), which describes parents' attitudes toward gender roles and the gender-based division of professions, and parental influence (PINF_mean), which indicates the level of parental influence on students' choice of profession. Both variables consist of several statements: PGA_mean has 8 (PGA1–PGA8), and PINF_mean has 6 (PINF1–PINF6). Both variables were measured on a 5-point Likert scale, with questions ranging from strongly disagree to strongly agree.

Data were collected using a specially designed structured questionnaire. The questionnaire consisted of several sections. In particular, parents' gender attitudes were measured using an 8-item scale (PGA1–PGA8). Parental influence on students' career choices was assessed using a 6-item scale (PINF1–PINF6). All statements were measured on a 5-point Likert scale, where 1 means “strongly disagree” and 5 means “strongly agree”.

To establish construct validity, the questionnaire items were systematically developed based on established theoretical frameworks, including Bourdieu's (1977) concept of “habitus” and social cognitive career theory. Prior to the main data collection, the survey instrument underwent rigorous content validity review by a panel of academic experts in sociology and educational psychology. This ensured that the items accurately captured the cultural nuances of parental gender attitudes and influence within the Kazakhstani context.

The reliability of the research instruments was assessed using internal consistency indices. In particular, Cronbach's alpha and McDonald's omega coefficients were calculated. The results showed high reliability for both scales: α = 0.885, ω = 0.885 for the parental gender attitudes scale, and α = 0.888, ω = 0.888 for the parental influence scale. These indices confirm the high internal consistency of the measurement instruments and the reliability of the research results.

Statistical analysis of the collected data was carried out using the JASP program. First, descriptive statistics were calculated to characterize the sample's socio-cultural profile. Then, Pearson's correlation analysis was used to assess the relationships among gender, cultural norms, and professional autonomy. Finally, linear regression analysis was conducted to predict the influence of traditional parental values on students' career choices.

## 4 Results

This section presents the statistical results of an empirical analysis examining the impact of parents' gender attitudes on students' professional choices. The analysis begins with a review of the participants' demographic profile and a description of the main variables. It then confirms the psychometric reliability of the measurement instruments. The section then presents the results of correlation and linear regression models used to test the hypotheses (H1, H2, and H3).

Table 2 presents the socio-demographic profile of the respondents who participated in the study (*N* = 701). The sample is dominated by women (65.0%) and men (35.0%), reflecting the gender disparity in Kazakhstan's higher education system, especially in social and humanitarian areas. The vast majority of respondents are first-year students (80.7%). This is a strategically important indicator for the study, since first-year students are in a transitional period from a family environment to an academic one, and their memories of parental influence when making a professional choice are the most relevant and authentic.

**Table 2:** Demographic characteristics of respondents (*N* = 701).

Variable	Category	*n*	%
Gender	Female	456	65.0
	Male	245	35.0
Year of study	1st year	566	80.7
	2nd year	95	13.6
	3rd year	29	4.1
	4th year	11	1.6
Major	Education	327	46.6
	IT/STEM	179	25.5
	Social Sciences	136	19.4
	Humanities	30	4.3
	Other	29	4.1
Influence source	Self	552	78.7
	Parents	122	17.4
	Teacher/Counselor	12	1.7
	Relatives	7	1.0
	Friends	3	0.4
	Other	5	0.7
Free choice	Yes	218	31.1
	No	343	48.9
	Not sure	140	20.0

Source: Compiled by the authors based on an online survey conducted in 2026.

The breakdown by professional field partially reflects the traditional gender segregation of professions in society. The largest group of respondents is educated in teaching professions (46.6%), followed by IT/STEM (25.5%) and social sciences (19.4%). This sample allows us to compare the dynamics of values between traditionally “feminine” fields and new technical fields.

The study revealed a significant social dissonance between subjective assessments and objective constraints regarding the process of choosing a profession. On the one hand, the absolute majority of students (78.7%) stated that they made a professional decision “on their own”, indicating that parental influence was direct in only 17.4% of cases. However, the assessment of the “level of freedom” of these respondents in choosing a profession showed a completely different picture: almost half (48.9%) admitted they did not have complete freedom in the decision-making process, and only 31.1% felt complete independence.

These demographic and descriptive data highlight the conflict between students' perceptions of individual choice and the constraints imposed by family culture. This trend underscores the need for a more in-depth statistical analysis to better understand the hidden influence of parental gender attitudes on young people's professional independence.

Table 3 presents descriptive statistics for the main variables of the study—parental gender attitudes (PGA_mean) and parental influence (PINF_mean; *N* = 701).

**Table 3:** Descriptive statistics of the main variables.

Statistic	PGA_mean	PINF_mean
Descriptive statistics		
Valid	701	701
Missing	0	0
Mean	2.998	2.671
Std. deviation	0.926	0.942
Minimum	1.000	1.000
Maximum	5.000	5.000

Source: Compiled by the authors based on an online survey conducted in 2026.

The average indicator of parents' gender attitudes (M = 2.998, SD = 0.926) is located exactly in the middle of the Likert scale. This statistical median is not a dry mathematical value, but an empirical reflection of the transitory socio-cultural state of modern Kazakhstani society. That is, the family values of the respondents are not completely traditional-patriarchal or completely egalitarian, but rather hybrid, combining elements of both systems. In addition, the high standard deviation (SD = 0.926) indicates a lack of consensus on gender norms within society and significant heterogeneity in cultural worldviews across families.

In turn, the level of parental influence on the student's choice of profession (M = 2.671, SD = 0.942) was slightly lower than average. From a socio-cultural point of view, this indicates young people's desire for formal autonomy in making professional decisions, but the large dispersion of the data (SD = 0.942) suggests polarization in family upbringing models. In some families, modern approaches emphasizing autonomy prevail, while in others, traditional parental authority and direct control remain strong.

The full range of both variables (1,000–5,000) confirms the diversity of respondents' social experiences. These descriptive results clearly indicate that gender attitudes and family influences are not just psychological factors, but rather a deep-seated value dichotomy. This fully justifies the need for a more in-depth analysis of the relationship between these variables using correlation and regression models.

Table 4 presents the internal consistency and psychometric stability of the main instruments measuring the variables. The calculation results showed that the scales “Parents' gender attitudes” (α = 0.885, ω = 0.885) and “Parental influence” (α = 0.888, ω = 0.888) have very high reliability. From the perspective of the methodology of socio-cultural research, reliability coefficients above 0.85 for both scales are an important indicator. This proves not only statistical stability but also the successful adaptation of the measuring instruments used to Kazakhstan's transit society.

**Table 4:** Reliability indicators of research scales.

Scale	McDonald's ω	Cronbach's α	95% CI lower	95% CI above
Gender perspective (PGA)	0.885	0.885	0.872	0.898
Parental influence (PINF)	0.888	0.888	0.876	0.901

Source: Compiled by the authors based on an online survey conducted in 2026.

Narrow 95% confidence intervals suggest that students across the study's demographic groups perceive these cultural constructs in a uniform, holistic way. Therefore, the empirical data collected can provide a solid methodological basis for conducting further complex correlational and regression analyses.

Table 5 presents the results of the Pearson correlation analysis between the main socio-cultural variables of the study—traditional gender attitudes of parents (PGA_mean) and the level of parental interference in students' professional autonomy (PINF_mean). The analysis revealed a statistically significant, moderately positive relationship between these variables (*r* = 0.427, *p* < 0.001). In the context of social sciences, the strength of such a relationship is considered quite high. From a socio-cultural perspective, this indicator is not merely a statistical coincidence but a direct empirical reflection of the mechanism of cultural reproduction in the family.

**Table 5:** Pearson correlation between variables.

Variable	PGA_mean	PINF_mean
Pearson's correlations		
**1. PGA_mean**		
Pearson's r	—	
*p*-value	—	
**2. PINF_mean**		
Pearson's r	0.427	—
*p*-Value	< 0.001	—

Source: Compiled by the authors based on an online survey conducted in 2026.

That is, the more rigid gender stereotypes and traditional roles of parents, the more proactively, and sometimes even directly, they become involved in the process of selecting a career path for their children.

This empirical fact fully supports the first hypothesis of the study (H1) and demonstrates that family gender norms are a decisive factor in determining the professional path of young people. The significant correlation identified shows that parents' values not only have an indirect but also a direct influence on professional orientation, confirming the validity of conducting a linear regression analysis to predict the impact of this phenomenon.

Table 6 presents the overall performance of the linear regression model, which tests the ability of parents' traditional gender attitudes to predict the level of interference in a student's professional choice. The results of the calculation showed that the constructed model is statistically significant at the 0.001 level (*p* < 0.001). Most importantly, this model explains 18.2% of the variance in parental influence (*R*^2^ = 0.182). Although this may seem like a “moderate” level by general mathematical standards, in the context of behavioral sciences and cross-cultural sociology, this is a very significant result.

**Table 6:** General indicators of the model.

Model	*R*	*R* ^2^	Adjusted *R*^2^	RMSE	*R*^2^ change	f1	df2	*p-*Value
Model summary—PINF_mean								
M_0_	0.000	0.000	0.000	0.942	0.000	0	700	
M_1_	0.427	0.182	0.181	0.852	0.182	1	699	< 0.001

M_1_ includes PGA_mean.

Source: Compiled by the authors based on an online survey conducted in 2026.

Given that dozens of exogenous and institutional factors influence a student's career choice, the fact that a single sociocultural variable accounts for nearly one-fifth (~18%) of the total variation in influence underscores the enormous determining power of family habitus. This quantitative indicator, therefore, demonstrates that gender norms in intergenerational communication are not merely a background phenomenon but a real structural force that directly influences young people's professional autonomy.

The results of the analysis of variance (ANOVA) presented in Table 7 clearly demonstrate the overall statistical significance of the constructed linear regression model (F_(1, 699)_ = 155.476, *p* < 0.001). Not only in a mathematical sense but also in a sociological sense, this very high F-criterion indicates that the influence of parents' traditional gender positions on the level of intervention in the student's professional choice is not a random phenomenon. From a socio-cultural perspective, this pattern of dispersion suggests that the mechanism for transmitting cultural norms in a transitioning society is highly structured and systematic.

**Table 7:** Results of analysis of variance (ANOVA).

Model	Sum of squares	df	Mean square	*F*	*p*-Value
ANOVA					
M_1_					
Regression	112.927	1	112.927	155.476	< 0.001
Residual	507.706	699	0.726		
Total	620.633	700			

M_1_ includes PGA_mean.

The intercept model is omitted, as no meaningful information can be shown.

Source: Compiled by the authors based on an online survey conducted in 2026.

In other words, parental control based on gender stereotypes is not an isolated occurrence in individual families, but rather a social institution that consistently operates within the population. This finding provides a solid scientific basis for understanding family pressure in the professional orientation process as an objective, structural force, rather than a collection of subjective opinions.

Table 8 presents the standardized and unstandardized coefficients of the linear regression model. The analysis showed that traditional gender views of parents have a direct, statistically significant effect on the level of intervention in the student's choice of profession (β = 0.427, *p* < 0.001). In the socio-cultural dimension, this standardized β (beta) coefficient is not just a mathematical proportion, but reflects the performative power of family cultural norms.

**Table 8:** Regression coefficients.

Model	Unstandardized	Standard error	Standardized	*t*	*p-*Value
Coefficients					
M_0_					
(Intercept)	2.671	0.036		75.100	< 0.001
M_1_					
(Intercept)	1.370	0.109		12.550	< 0.001
PGA_mean	0.434	0.035	0.427	12.469	< 0.001

Source: Compiled by the authors based on an online survey conducted in 2026.

That is, the higher the level of gender stereotypes among parents, the more likely they are to proactively intervene, even directly, in the professional choices of young people. This indicator shows that traditional gender beliefs are not just passive ideas, but become concrete actions during the intergenerational transfer. Thus, the data fully support the study's main hypotheses, confirming that gender segregation is reproduced primarily at the micro level through family influence. Overall, the comprehensive analysis conducted proves that all three hypotheses proposed in the study's conceptual framework are statistically supported.

First, hypothesis H1, based on Bem (1981) gender schema theory, was confirmed: a significant positive relationship (*r* = 0.427, β =0.427, *p* < 0.001) was found between parents' traditional gender attitudes and the level of parental involvement in the process of choosing a profession for students. Second, the regression model confirmed hypothesis H2, showing that in families with a dominant “conservation” value, parents tend to direct young people toward traditional professions, regardless of the requirements of the modern labor market. Third, the disproportionate distribution of professional areas confirmed hypothesis H3, based on Bourdieu's (1986) concept of social capital. It was found that traditional gender habitus in the family significantly limits the autonomy of girls to choose STEM fields more than boys. That is, the majority of female respondents were concentrated in traditional fields such as pedagogy.

Thus, the obtained quantitative results show that parents with strong gender stereotypes are more directly involved in the professional choices of their children. This empirical data shows that the process of choosing a profession is not a purely psychological act but a socio-cultural construct influenced by family gender attitudes and cultural values.

## 5 Discussion

The empirical results obtained in this section are analyzed to better understand the complex relationship between parental gender attitudes and students' professional choices in a socio-cultural context. Quantitative indicators are discussed in relation to fundamental scientific theories regarding the transformation of macro-social values and the intersection of family gender stereotypes.

The main empirical paradox of the study was reflected in the subjective assessment of students' professional autonomy. According to the demographic data (Table 2), although 78.7% of respondents stated that they chose their profession independently, 48.9% admitted they did not have complete freedom in the process. This statistical dichotomy should not be interpreted as a survey bias, but rather as direct empirical evidence supporting Bourdieu's (1986) theory of social capital and the internalization of social structures.

In Bourdieu's (1986) theory, this contradiction creates the “illusion of personal choice”. Habitus acts as a social construct that unknowingly limits an individual's range of possibilities while maintaining their sense of freedom to make decisions. This is because, although students believe they independently choose their career path, their choices are pre-filtered by traditional gender roles and social norms transmitted by their parents regarding which professions are considered prestigious, socially acceptable, and accessible.

Critically, this suggests that young people's career orientation is not based on a neoclassical rational-choice process but rather on an invisible framework of family and cultural capital. The majority of female respondents in the sample, who are concentrated in “feminine” fields such as pedagogy (46.6%), is a direct consequence of this unconscious social filter. This trend not only demonstrates young people's reluctance to consider gender-inconsistent alternatives but also shows how structural gender segregation in the education system is perpetuated at the family level.

The macro-social dimension of the study is reflected in the mean value of parents' gender attitudes (PGA_mean = 2.998; Table 3). This median indicator on a five-point scale indicates that gender attitudes in Kazakhstani families are not in a strictly patriarchal or fully egalitarian polarity, but rather in a hybrid cultural state. This empirical fact directly corresponds to Inglehart and Baker's (2000) theory of modernization. As Inglehart and Baker (2000) argues, the economic society is undergoing a transition from “survival” values to “self-expression” orientations. This period, from a socio-cultural perspective, creates a dichotomy of values in Kazakhstan. Parents consider higher education for their daughters to be mandatory, in line with the demands of globalization and economic competition. However, they try to limit their daughters' professional choices to traditional, “safe” fields that align with family values.

Crucially, this hybrid state also puts male students under pressure. While girls are steered away from STEM subjects toward “safe” professions, boys often experience immense pressure to avoid fields deemed “feminine” by society, such as primary education or nursing. Instead, they are encouraged to pursue financially rewarding technical or managerial roles that fulfill the traditional expectation of the male breadwinner, demonstrating that cultural stereotyping restricts the professional autonomy of both genders in different ways.

This phenomenon also has a close conceptual connection with Schwartz's (1992) theory of basic human values. The statistical significance of parental influence (PINF_mean =2.671) confirms the dominant role of “preservation” and security orientations in the family decision-making process in the model of Schwartz (1992). Compared with individualistic models in Western countries, parental authority and economic pragmatism remain key socio-cultural factors in the post-Soviet space that limit students' professional autonomy. Accordingly, family involvement in choosing a profession is not merely a matter of care but a strategic social control mechanism designed to preserve cultural capital and maintain adherence to social norms.

The results of linear regression and correlation analysis (Tables 4, 7) confirmed that the influence of parents' gender views on student choice is very strong (*r* = 0.427, β = 0.427, *p* < 0.001). This pattern fully justifies Behm's (1981) gender schema theory at the micro-level. The manifestation of traditional gender roles among parents leads to their active influence on their children's professional choices. This, in turn, strengthens young people's internalization of these cognitive schemes and their perception of professions through a gender prism (Weisgram and Diekman, 2017). Thus, hypotheses H1 and H2 were fully confirmed empirically.

Career choice is not a one-time decision, but a continuous process that reinforces gender norms through daily communication within the family. The family plays a significant role in shaping gender hierarchies by either supporting or discouraging a child's interest in certain career paths (Van de Rozenberg et al., 2024). Disapproval of girls' aspirations to pursue IT or skepticism about boys' choices to study teaching are examples of these influences. This process contributes to gender segregation in science, technology, engineering, and mathematics (STEM) fields, leading to girls' underrepresentation in technical careers (Alam, 2022).

This performativity is also closely linked to Bandura's (1986, 1997) social cognitive theory and Roe's (2008) career choice theory. Gender expectations and parental emotional support shape students' sense of self-efficacy and career motivation (Chan, 2022; Lv et al., 2022). As a result, students rely not only on their own abilities but also on the support and encouragement of their families in pursuing their chosen career. This factor significantly influences their professional ambitions, particularly in non-traditional fields (Kelly, 2016).

According to the regression analysis (Table 6), parents' gender attitudes account for 18.2% of the variance in students' choice of major (*R*^2^ = 0.182). Despite the presence of many exogenous factors typical of the behavioral sciences (peers, media, macroeconomics), the high explanatory power of a single socio-cultural variable confirms the decisive influence of the family institution. This mechanism needs to be examined in more detail on the basis of social-cognitive theory and theories of major choice.

Specifically, according to social cognitive career theory (SCCT), parental gender stereotypes are not imposed on young people in a deterministic or mechanistic way. Rather, they are filtered through the individual's internal cognitive and psychological processes. In this process, parental disapproval or skepticism can act as a negative social influence, reducing the student's self-confidence and expectations for a specific career path (Chan, 2022; Lv et al., 2022). From a career choice perspective, these family reactions narrow the range of acceptable career options for adolescents along gender lines. In societies with strong intergenerational connections, this cognitive evaluation process can be complicated by norms of respect for family authority and duty. As a result, students must balance their personal career interests with family expectations.

Thus, during career orientation, students evaluate not only their true academic abilities, but also the level of family and social approval of their chosen profession. This pragmatic compromise pushes young people, especially girls, away from highly profitable but socially “masculine” fields such as STEM, and toward socio-humanitarian directions where family support is guaranteed (Smith and Wood, 2020; Tandrayen-Ragoobur and Gokulsing, 2021). Therefore, choosing a profession is not just an individual motivational act, but a complex socio-cultural compromise that is carried out through the cognitive adaptation of family values and gender norms. Based on these mechanisms, the hypothesis H3 that traditional gender roles in the family limit the autonomy of girls to choose a profession (especially in technical/STEM directions) more than boys is fully confirmed.

The place of residence indirectly influences students' choice of major through parental attitudes toward gender. In cities, there are more flexible attitudes toward gender roles, which allows for the choice of diverse and prestigious educational trajectories, including non-traditional majors. In rural areas, traditional gender norms are more strongly maintained, and gender stereotypes often influence the choice of major.

These differences are associated with regional educational infrastructure and socio-economic conditions: rural youth are more likely to choose professions available in large cities, and their choices are often determined by economic constraints. As a result, gender attitudes and material opportunities interact to shape students' professional orientation.

Family income and parental gender roles play a significant role in determining a person's choice of profession. In high-income families, children have more opportunities to pursue interests outside traditional gender roles. However, in low-income families, traditional gender expectations and economic factors often dominate, leading to a more limited range of choices. Therefore, choosing a career is not solely an individual decision but rather a process shaped by one's family's socio-cultural and value systems.

The discussion highlighted the complex and multidimensional nature of family influences and gender stereotypes. However, it also had some methodological limitations. Firstly, the cross-sectional design of the study does not allow us to fully understand the long-term causal relationships between gender attitudes and professional positions. Secondly, as the study relied primarily on convenience sampling among first-year students in southern Kazakhstan, it is necessary to exercise caution when generalizing these findings nationwide. First-year students are in a transitional phase where family influence is at its strongest, so their perspectives may change by the time they graduate. When viewed through the theoretical lens of Bourdieu's (1977) concept of habitus, the high concentration of freshmen suggests that their current career choices are more likely to be reflections of their parents' deeply ingrained habitus than of their own, fully developed professional identity. Thirdly, as most respondents were female and from a traditionally “feminine” field of education, the experiences of male students and the influence of family pressures on their professional choices were not fully explored.

Finally, relying on self-reported data means there is a possibility that students may subconsciously exaggerate their independence. This may partly explain why parental influence accounted for 18.2% of the variance. This reminds us that peers, educators and labor market realities also play significant roles.

Moreover, the model's relatively low explanatory power requires us to reflect critically on unmeasured exogenous factors. Specifically, the model did not include crucial variables such as socioeconomic status, academic achievement and individual interests. This suggests that, while family capital serves as an important initial filter, the remaining variance in students' professional decision-making could be accounted for by these missing variables.

To overcome the limitations of existing research on the topic, future studies should adopt a longitudinal design. This method would allow for measuring students' cultural and professional development over time, from their initial coursework to their integration into the job market. Additionally, a mixed-methods approach, combining quantitative and qualitative data, would be essential. Qualitative research provides a unique opportunity to gain insight into the micro-level processes of “generation” in Kazakh families, including ethno-cultural characteristics and strategies for overcoming parental habits among young people.

## 6 Conclusion

This study comprehensively explored the socio-cultural factors underlying gender segregation in students' career choices in the context of globalization and the radical transformation of the labor market. The empirical analysis confirms that professional choice is not merely a psychological act but a complex social construction, guided primarily by traditional gender roles and the cultural expectations of parents. It has been demonstrated that the persistence of gender stereotypes within families directly limits the professional autonomy of young people, serving as a key factor in their orientation toward socially acceptable “safe” fields in society.

The main theoretical contribution of this study is the scientific justification of the hybrid nature of value exchange between generations in Kazakh society. We have found that the concept of “indirect gender bias”, which is often found in Western scientific literature, is transformed into direct pressure in the Kazakh context to internalize parental authority and family structures. This suggests that young people's professional choices are shaped by economic pragmatism and parental supervision.

In this regard, the research findings call for a fundamental revision of the national career guidance paradigm. Moving away from traditional individualistic approaches and toward a “family-centered” inclusive format is an urgent strategic need. This is because it is impossible to change a student's professional choice in isolation from the family habitus surrounding them. State education policy should prioritize deconstructing entrenched gender stereotypes in the minds of parents and raising their awareness of the demands of the new labor market. At the institutional level, psychologists and educators need to develop comprehensive interventions to strengthen young people's sense of self-efficacy and alleviate the cognitive dissonance between family expectations and personal professional ambitions.

The study showed that overcoming structural gender segregation in the labor market and fully realizing young people's human capital are not limited to formal economic or educational reforms. The crux of the problem lies in the continuous synchronization between macro-level institutional changes and micro-level family cultural transformation. To create an innovative economy in Kazakhstani society and achieve a new stage of personal and professional freedom, it is necessary, first of all, to liberalize gender dogmas in intergenerational value exchange. Institutionally protecting and developing students' autonomy of choice is not just a matter of social justice; it should become a key value that enhances the nation's global competitiveness.

Building upon these findings, future research must address several profound, open-ended questions concerning the intersection of future work and family dynamics. For example, if society moves toward a “family-oriented” approach to career counseling, researchers must explore how to balance the need for individual career freedom with a deep respect for cultural traditions that prioritize family unity.

Furthermore, as the Kazakhstani labor market becomes more globalized, it is important to investigate whether the “invisible framework” of family capital will eventually weaken or whether parents will simply find new, more subtle ways to encourage their children to pursue “safe” roles. Finally, longitudinal studies should examine the long-term emotional cost to students who choose gender-inconsistent paths.

Understanding whether a lack of psychological support from their family hinders their professional success later in life, regardless of their talent, would elevate this line of enquiry from a data exercise to a vital examination of the evolving heart of society.

## Data Availability

The raw data supporting the conclusions of this article will be made available by the authors, without undue reservation.
